# Serum Otoconin-90 and Otolin-1 Concentrations in Benign Paroxysmal Positional Vertigo

**DOI:** 10.3390/biom14101279

**Published:** 2024-10-10

**Authors:** Demet Aygun, Seyma Dumur, Mehmet Nuri Elgormus, Mehmet Serkan Alpaslan, Hafize Uzun

**Affiliations:** 1 Department of Neurology, Faculty of Medicine, Istanbul Atlas University, 34408 Istanbul, Turkey; demetaygun@yahoo.com; 2 Department of Medical Biochemistry, Faculty of Medicine, Istanbul Atlas University, 34408 Istanbul, Turkey; seyma.dumur@atlas.edu.tr; 3 Department of Otorhinolaryngology, Faculty of Medicine, Istanbul Atlas University, 34408 Istanbul, Turkey; mehmetnuri.elgormus@atlas.edu.tr (M.N.E.); mehmet.alpaslan@atlas.edu.tr (M.S.A.)

**Keywords:** benign paroxysmal positional vertigo, otolith-associated protein otoconin-90, otolin-1, vitamin D

## Abstract

Objective: The aim was to evaluate the value of otolith-associated protein otoconin-90 (OC90) and otolin-1 in the pathogenesis research and clinical treatment of benign paroxysmal positional vertigo (BPPV). Material and Method: The study included 50 patients with BPPV admitted to neurology and otorhinolaryngology departments and 30 healthy subjects with no history of dizziness as a control group. Results: BPPV and controls were similar in terms of gender and age. Otolin-1 concentration was significantly greater in the BPPV group than in the controls (710.44 [584.35–837.39] vs 280.45 [212.7–419.61]; *p* < 0.001). No statistical significance was found, although OC90 was higher in the BPPV group than in the controls. There was a strong positive correlation between otolin-1 and OC90, a moderate negative correlation between otolin-1 and vitamin D, and a strong negative correlation between OC90 and vitamin D in the BPPV patient group. Otolin-1 had high specificity and AUC values for BPPV (AUC: 0.933; 95% CI: 0.881–0.986, 79.2% sensitivity, 100% specificity with a cutoff greater than 525). Conclusions: High serum concentrations of otolin-1 were associated with an increased risk of BPPV, but high concentrations of OC90 were not. Serum concentrations of otolin-1 can potentially be used as a biomarker for the acute onset of inner ear disorders due to the significant increase in patients with BPPV. Vitamin D has high specificity and sensitivity in patients with BPPV. It also provides evidence that BPPV patients with vitamin D deficiency may improve their symptoms with replacement therapy. More large-scale prospective studies are required to confirm these associations and clarify the exact mechanisms.

## 1. Introduction

Benign paroxysmal positional vertigo (BPPV) is one of the most common causes of dizziness. Peripheral vestibular disorders are a diverse group of diseases characterized by vestibular symptoms that may be acute, episodic or permanent [1,2]. The most common peripheral vestibular disorders are BPPV and Meniere’s Disease, and others are vestibular neuritis, bilateral vestibulopathy, vestibular paroxysm and superior canal dehiscence syndrome [2,3].

Due to the complexity of the etiopathogenesis and clinical presentation of peripheral vestibular diseases, their diagnosis and treatment can be challenging for the clinician, and prognosis can sometimes be difficult to predict [4]. In general, these diseases are diagnosed by history, vestibular examination and tests, but there is no systemic biomarker specifically related to the inner ear [5]. Otolin-1 is a glycoprotein whose mRNA expression is limited to the inner ear. It is specifically produced by marginal cells in the vestibular macula, semicircular canal crystal, organ of Corti and stria vascularis. Its main function is to interact with other inner-ear-specific proteins such as otolith-associated protein otoconin-90 (OC90) and to form otoconia [6,7,8]. Therefore, otolin-1 protein is a serologic biomarker recently defined in the literature as specific for the inner ear [6]. Otolin-1 is specific for the inner ear and can be easily detected in serum as it crosses the blood–brain barrier, and its concentration increases in correlation with pathology [9]. In this sense, it may be used as an ideal biomarker in inner ear pathologies and specifically in peripheral vestibular diseases.

During otolith development, the organic matrix is formed before calcium carbonate deposition. Collagen OC90 is required for otolith formation [10]. OC90 is the main matrix protein of the mammalian calcifying otolith; it retains the ability to bind calcium and is highly acidic so that calcium or CaCO_3_ can bind. In the absence of OC90, matrix-bound calcium on the surface of the vestibular macula is greatly reduced [11].

However, to our knowledge, no study investigating the association of serum OC90 and otolin 1 concentrations with BPPV has been published yet. The aim of this study was to evaluate the systemic specific biomarkers that may help clinicians in BPPV; that may be included in the diagnostic, treatment and follow-up criteria; that may help explain the etiopathogenesis of peripheral vestibular disorders; that may help differentiate them from central vestibulopathies; and that may also be used for different inner ear pathologies in the light of the literature.

## 2. Material and Method

### 2.1. Ethical Approval

Permission was obtained from the Clinical Research Ethics Committee of Istanbul University Cerrahpasa, Cerrahpasa Medical Faculty (Date: 22 April 2024, Code: E-74555795-050.04-971548), before the study began. This study was conducted according to the Declaration of Helsinki. All subjects gave their informed consent for inclusion before they participated in the study. All subjects were of Turkish descent.

### 2.2. Research Design

This is a cross-sectional study conducted in the outpatient clinics of the neurology and otorhinolaryngology departments of Medicine Hospital, Medical Faculty, Istanbul Atlas University. A total of 80 participants aged 21–62 years were recruited for this study. Our study involved 50 consecutive BPPV patients (26 females and 24 males) in the departments of neurology and otorhinolaryngology, and 30 age- and sex-matched controls (17 females and 13 males).

All participants underwent audiometric examination after routine neurotologic examination.

### 2.3. Inclusion Criteria

Patients diagnosed with BPPV were included in this study. The diagnosis of BPPV was confirmed by considering the history of dizziness and the character of nystagmus during the Dix–Hallpike maneuver. The Epley maneuver was performed in all patients after the affected side was determined by considering the channel indicated by the nystagmus. The patients were evaluated two or three days later, and the maneuvers were repeated. The Dix–Hallpike maneuver is the gold standard for diagnosing BPPV in vertical-semicircular canals. The maneuvers were continued until no nystagmus was observed in the Dix–Hallpike test [12].

In the study, 30 healthy individuals with no known chronic diseases, who were not taking any medication and were aged between 18 and 62 years, were recruited as the control group. The control group consisted of patients who presented to our clinic with complaints other than dizziness, tinnitus or imbalance, who had serum vitamin D concentration measurements and who had not consulted a physician for vertigo, dizziness or imbalance in the year before presentation.

### 2.4. Exclusion Criteria

Subjects with pathology on neuro-otologic examination, unexpected hearing loss on audiometric examination, history of inner ear disease (Meniere’s disease, autoimmune inner ear disease, vestibular neuritis, acoustic neurinoma, sudden hearing loss, temporal bone fracture, drug-induced ototoxicity, etc.), history of head trauma, history of peripheral vestibular disease, history of otologic surgery, chronic systemic disease (such as hypertension, hyperlipidemia, hypothyroidism, hyperparathyroidism, diabetes mellitus or cardiac diseases) pregnancy, vitamin D replacement therapy for osteoporosis or osteopenia were excluded.

### 2.5. Sample Collection and Measurements

The blood samples were collected in 2 mL K3-EDTA blood collection tubes for complete blood count (CBC) and 5 mL anticoagulant-free tubes (Becton, Dickinson and Company, Franklin Lakes, Bergen, NJ, USA) for OC90, otolin-1, and routine biochemical parameters after an overnight fast (≥8 h). Serum samples were obtained after at least 30 min of clotting by centrifugation at 2500× *g* for 15 min and stored at −80 °C until OC90 and otolin-1 could be studied. Routine biochemical parameters and 25-hydroxyvitamin D3 (25(OH)D3, Vit D3) were measured on the day of blood collection.

CBC was recorded with an automatic hematology analyzer (Sysmeks XN-1000, Norderstedt, Germany). The biochemical parameters were measured using the spectrophotometric method with an automated biochemistry analyzer (Architect i2000, Abbott Park, IL, USA). The serum CRP concentrations were measured using the nephelometric method (Immage 800 Beckman Coulter, Brea, CA 92821, USA). CRP analysis was performed at the time of admission. The serum 25-hydroxyvitamin D [25(OH)D] concentrations were measured by enzyme-linked fluorescent assay on the Mini Vidas (Biomerieux, Paris, France).

### 2.6. Measurement of Serum Otoconin 90 (OC90) Concentrations

OC90 concentrations were measured (twice) according to the manufacturer’s instructions (Human Otoconin 90 ELISA Kit, Cat. No.: E2535Hu, BT LAB, Zhejiang, China). The plate was pre-coated with human OC90 antibody. OC90 present in the sample was added and bound to antibodies coated on the wells. Then, biotinylated human OC90 antibody was added and bound to OC90 in the sample. Then, Streptavidin-HRP was added and bound to the Biotinylated OC90 antibody. After incubation, unbound Streptavidin-HRP was washed away during a washing step. Substrate solution was then added, and the color developed in proportion to the amount of Human OC90. The reaction was terminated by the addition of acidic stop solution. Absorbance was measured at 450 nm. Results were obtained using Biotek Elx800 (Santa Clara, CA, USA) ELISA equipment. The coefficients of intra- and interassay variation were <8% (*n* = 20) and <10% (*n* = 20), respectively.

### 2.7. Measurement of Serum Otolin-1 Concentrations

Otolin-1 concentrations were measured according to the manufacturer’s instructions (Human Otolin 1 ELISA Kit, Cat. No.: E3947Hu, BT LAB, Zhejiang, China). The plate was pre-coated with human otolin-1 antibody. Otolin-1 present in the sample was added and bound to antibodies coated on the wells. Then, biotinylated human otolin-1 antibody was added and bound to otolin-1 in the sample. Then, Streptavidin-HRP was added and bound to the biotinylated otolin-1 antibody. After incubation, unbound streptavidin-HRP was washed away during a washing step. Substrate solution was then added, and the color developed in proportion to the amount of human otolin-1. The reaction was terminated by the addition of acidic stop solution and absorbance was measured at 450 nm. Results were obtained using Biotek Elx800 (Santa Clara, CA, USA) ELISA equipment. The coefficients of intra- and interassay variation were <8% (*n* = 20) and <10% (*n* = 20), respectively.

### 2.8. Statistical Analysis

The Statistical Package for the Social Sciences version 21.0 software package for Windows (IBM Corp., Armonk, NY, USA) and JASP 0.16.4.0 were used for data evaluation and analysis. Categorical variables are presented as frequencies (n) and percentages (%), and numerical variables are presented as mean ± standard deviation or medians (25th percentile–75th percentile). Whether the data were normally distributed was analyzed through visual (histograms and Q–Q plots) and descriptive (coefficient of variation, skewness and kurtosis) techniques and analytical methods (Kolmogorov–Smirnov test). The independent samples *t*-test or Mann–Whitney U-test was used to compare continuous variables between two independent groups. Spearman correlation analysis was used to evaluate the relationships between the numerical variables. ROC analysis was applied to evaluate the cutoff values, sensitivity and specificity of the parameters. A value of *p* < 0.05 was considered to indicate statistical significance.

## 3. Results

In the present study, 54.17% of the patients and 65.38% of the controls were female; the gender distributions of the case and control groups were similar (*p* = 0.350). The mean age of the case group was 42.46 ± 11.4 years, while the mean age of the control group was 38.69 ± 11.2 years, and there was no significant difference between them (*p* = 0.176). All the patients had dizziness, while 29.17% of the patients had tinnitus (35.71%-right; 64.29%-left). Symptoms were present within the last week in 29.17%, 1–3 weeks in 54.17%, and for more than 4 weeks in 16.67% of the patients. Etiologically, 50% of the patients were idiopathic, 40% experienced symptoms after upper respiratory tract infection (URTI), 4% (*n* = 2) had traumatic etiology, and 4% (*n* = 2) had a pressure difference. None of the patients had hearing loss on audiological examination. Nystagmus was present in 39.58% of the patients (Table 1).

No statistically significant differences were observed between the case and control groups in terms of calcium, parathormone (PTH), IG%, WBC, RBC, HGB, HCT, MCV, MCH, MCHC, PDW, PCT, RDW-SD, lymphocytes, lymphocytes%, monocytes, monocytes%, monocytes, eosinophils, eosinophils% or the SII.

**Figure 1:**
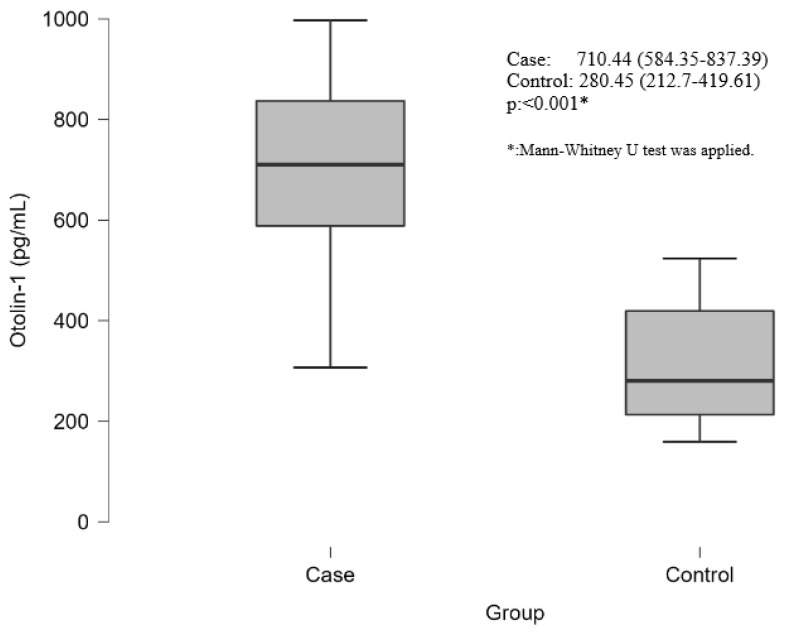
Box plot graph of otolin-1 concentration differences between patient and control groups.

The vitamin D concentration was significantly lower in the case group than in the control group (14.3 (8.45–17.5) vs. 26.25 (21–32); *p* < 0.001). CRP concentrations were significantly greater in the case group than in the control group (3.3 (2–5.35) vs. 1.18 (0.68–1.7); *p* < 0.001). The NLR was significantly greater in the case group than in the control group (1.66 ± 0.52 vs. 1.29 ± 0.37; *p* < 0.001). In addition, the neutrophil and neutrophil percentage were greater in the case group than in the control group. The PLR was significantly lower in the case group than in the control group (78.04 (62–94.74) vs. 98 (85.6–117.96); *p* = 0.003). In addition, the platelet count was lower in the case group than in the control group (Table 2). Otolin-1 concentrations were significantly greater in the case group than in the control group (710.44 (584.35–837.39) vs. 280.45 (212.7–419.61); *p* < 0.001) (Table 3, Figure 1). No statistical significance was found, although OC90 concentrations were greater in the case group than in the control group (Table 2).

**Figure 2:**
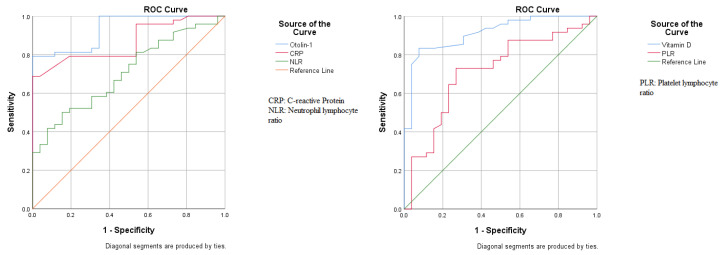
ROC analysis of hematological parameters for benign paroxysmal positional vertigo patients.

Table 4 and Figure 2 present the ROC analysis results for BPPV. The AUC was 0.933 (95% CI: 0.881–0.986), with 79.2% sensitivity and 100% specificity, with a cutoff value greater than 525 for otolin-1. The AUCs were 0.867 (95% CI: 0.787–0.947) for CRP, 0.705 (0.587–0.823) for NLR, and 0.713 (0.588–0.838) for PLR. The AUC was 0.913 (0.848–0.979), with 83.3% sensitivity and 88.5% specificity, with a cutoff of less than 20 for vitamin D.

In all study groups, otolin-1 was weakly positively correlated with OC90 (r = 0.393; *p* = 0.001) and strongly negatively correlated with vitamin D (r = −0.682; *p* < 0.001). OC90 was moderately negatively correlated with vitamin D (r = −0.404; *p* < 0.001). Similarly, there was a significant correlation between all three parameters in the case group. There was a strong positive correlation between otolin-1 and OC90 (r = 0.693; *p* < 0.001), a moderate negative correlation between otolin-1 and vitamin D (r = −0.586; *p* < 0.001) and a strong negative correlation between OC90 and vitamin D (r = −0.672; *p* < 0.001). In contrast, there were no correlations between the three parameters in the control group (*p* > 0.05) (Table 5).

## 4. Discussion

In the present study, serum OC90, otolin-1 and vitamin D concentrations of BPPV patients and healthy subjects without vestibular complaints were compared. While serum otolin-1 concentrations were found to be high, vitamin D concentrations were found to be low in BPPV patients. Although the serum concentration of OC90, the major soluble matrix protein of otoconia, was observed to be higher in BPPV patients than in control subjects, no statistically significant difference was identified. There was a strong positive correlation between otolin-1 and OC90, a moderate negative correlation between otolin-1 and vitamin D, and a strong negative correlation between OC90 and vitamin D in the BPPV patient group. Vitamin D has high specificity and sensitivity, while otolin-1 has high specificity and low sensitivity. Circulating otolin-1 and vitamin D can be used as a serum biomarker to assess the diagnosis in BPPV. In patients with BPPV, recurrence can be reduced by bringing serum vitamin D concentrations to normal concentrations with replacement therapy.

Although there is no conclusive evidence about the etiology of BPPV, advanced age, head trauma, various diseases affecting the inner ear, female gender, hormonal factors, a viral or ischemic cause, hyperlipidemia, osteoporosis, vitamin D deficiency and familial predisposition are blamed [13]. In the present study, there was no difference between the groups in terms of age and gender. Overall, 52% of the patients were idiopathic, 40% experienced symptoms after upper respiratory tract infection (URTI), 2% had traumatic etiology and 2% had a pressure differential. The presence of a previous infection is evidence in favor of a viral etiology. It has been reported that more than 50% of patients with BBPV have an idiopathic form [14]. Our results are consistent with the literature.

Otoliths are crystalline structures composed of calcium carbonate and glycoproteins. The mechanism of BPPV is explained by otoliths such as OC90 and otolin-1 that break away from the otolithic membrane and escape into the semi-circular canals [15]. In the present study, serum OC90 concentrations were greater in BPPV than in the control group. However, the difference in the serum OC90 values between the two groups was not found to be statistically significant. Bi et al. [16] found that otoconin-90 concentrations in the peripheral blood of patients with BPPV were significantly higher than those in healthy controls. The blood concentrations of OC90 also showed a high positive correlation with age. The results reflect the process of otoconia degradation with age. Zhao et al. [10] demonstrated that OC90-knockout mice do not effectively recruit Ca^2+^ to the macula from the bloodstream during development and have rod-like large calcitic aggregates that are susceptible to dissolution. In contrast to our study, Zhank et al. [17] reported that patients with BPPV compared with in controls had notably lower plasma concentrations of OC90. The reason for the lack of significant difference between the OC90 concentrations of the patient and control groups in our results is either inadequate study population or insufficient passage of the protein from the tissue to the systemic circulation. For example, otolin-1, an otolith protein, is specifically expressed in the inner ear but can be detected in serum when it moves into the systemic circulation via the blood–labyrinth barrier [6,18]. Further investigation is required to determine how the concentration of inner-ear-associated proteins such as OC90 change in the systemic circulation.

The concentrations of otolin-1 in systemic circulation increase with age, while BPPV increases in prevalence with age [19,20,21,22,23]. In the present study, otolin-1 concentrations were significantly greater in the BPPV group than in the control group. Parham et al. [18] reported that even though the mean serum otolin-1 concentration was significantly higher in patients with BPPV, absolute concentrations of serum otolin-1 were only higher than the control group in one third of the postmenopausal BPPV patients. The authors explained this disparity as possibly being due to the enrollment of the subjects up to two years from the BPPV episode. Wu et al. [24] determined that serum concentrations of the otolin-1 protein were significantly higher in patients with BPPV than in healthy controls and may serve as a potential biomarker for BPPV episodes and could be used to promote better management of BPPV clinically. Both parathyroid hormone (PTH) and total calcium concentrations affect otolin-1 concentrations, implying that the calcium dysregulation caused by primary hyperparathyroidism may contribute to the otoconia breakdown and may be associated with inner ear disorders such as BPPV [25]. In a recent study, in the same way, the serum concentrations of otolin-1 in patients with BPPV were significantly higher compared with individuals without BPPV [26]. Elevated serum concentrations of otolin-1 were also associated with an increased risk of recurrent BPPV [9]. The meta-analysis indicated that there is a higher serum concentration of otolin-1 in patients with BPPV than in healthy controls. Therefore, otolin-1 may serve as a biomarker for the onset of BPPV [27]. Contrary to the above findings, the serum concentrations of otolin-1 were not significantly different between patients with Meniere disease (MD) in the interictal phase and the control group’s healthy ones [28]. Our results suggested that serum otolin-1 concentrations may serve as a biomarker for BPPV episodes.

Calcium channel proteins involved in calcium metabolism in vestibular end organs have been shown to be vitamin D-dependent. Studies on this subject have shown that rats lacking vitamin D receptors in vestibular cells develop vestibular dysfunction [29,30]. In the present study, the vitamin D concentration was significantly lower in the BPPV patients than in the control group. There was a strong positive correlation between otolin-1 and OC90, a moderate negative correlation between otolin-1 and vitamin D, and a strong negative correlation between OC90 and vitamin D in the BPPV patients. Jeong et al. [31] found that serum 25-OH vit D concentrations were lower in 100 patients with BPPV compared to the control group (*n* = 192), and that vitamin D deficiency and osteoporosis were risk factors that could significantly affect BPPV recurrence in these patients. Büki et al. [32] reported that serum vitamin D concentrations were lower in patients with BPPV recurrence compared to patients without recurrence, and no recurrence was reported when vitamin D replacement was performed in these patients. Talaat et al. [33] reported that vitamin D replacement therapy had positive effects on BPPV recurrence. Similarly, it has been reported that in patients with recurrent BPPV who are under rehabilitation therapy, raising serum 25-OHD to normal values reduces the recurrent rate of BPPV significantly [34]. In a study involving 232 patients diagnosed with BPPV, vitamin D deficiency was found to be a risk factor for the development of BPPV independent of age, gender and type of BPPV [35]. Karatas et al. [36] reported that there was no association between BPPV and vitamin D deficiency, and vitamin D deficiency is also not risk factor for BPPV. The results of studies on vitamin D in patients with BPPV are controversial [35,36,37,38,39,40,41]. Therefore, more large-scale prospective studies are needed.

### Limitations of Study

Our study has some limiting factors. (i) The small number of patients in the study. (ii) Recurrence patients were not included in the study. (iii) Patients with vitamin D deficiency who received replacement therapy were not included in the study. (iv) Epely manevrasından sonra biyokimyasal parametrelere bakılmadı (Oc90, otolin-1, Vitamin D).

## 5. Conclusions

The present study reveals that the serum concentrations of OC90 are not significantly different between the patients with BPPV and the healthy controls. High serum concentrations of otolin-1 were associated with an increased risk of BPPV. Serum concentrations of otolin-1 can potentially be used as a biomarker for the acute onset of inner ear disorders due to its significant increase in patients with BPPV. Vitamin D concentration was significantly lower in the BPPV patients and had high specificity and sensitivity in the patients with BPPV. The study also provides evidence that BPPV patients with vitamin D deficiency may improve their symptoms with replacement therapy. There was a significant correlation between all three parameters in the BPPV patients. The otoconia organic matrix is composed of a variety of proteins, the chief constituents of which are otoconin-90 and otolin-1, but additional work is needed to establish their value and clarify the exact mechanisms. The evaluation of serum biomarkers of CO90 and otolin-1, including vitamin D, in a larger cohort of patients with a clinical diagnosis of BPPV, may help to further elucidate the role of otolithic biomechanical disruption. The evaluation of serum biomarkers, an evolving area of research with the potential to reveal the pathophysiological mechanisms underlying various inner ear pathologies, will be included in our future projects.

## Figures and Tables

**Table 1:** Demographic characteristics and clinical signs and symptoms of patients in the case and control groups.

	Case		Control		
	n	%	n	%	*p*-Value
Gender					
Male	24	45.83%	13	34.62%	0.350 *
Female	26	54.17%	17	65.38%	
Age (mean ± std)	42.46 ± 11.4		38.69 ± 11.2		0.176 ^†^
Tinnitus	14	29.17%			
Right	5	35.71%	-	-	-
Left	9	64.29%	-	-	
Duration					
<1 week	14	29.17%	-	-	-
1–3 week	26	54.17%	-	-	
≥4 week	8	16.67%	-	-	
Cause					
Idiopathic	26	52%	-	-	-
After the URI	20	40%	-	-	
Traumatic	2	4%	-	-	
Pressure difference	2	4%	-	-	
Neurological examination					
Normal	30	60.00%	-	-	-
Nystagmus	20	40.00%	-	-	

URTI, upper respiratory tract infection. *: Chi-square test; ^†^: Independent samples *t*-test.

**Table 2:** Comparison of laboratory findings between the case and control groups.

	Case	Control	
	Mean ± Std or Median (25 p–75 p)	Mean ± Std or Median (25 p–75 p)	*p*-Value
Otoconin-90 (ng/L)	655.13 (553.47–1505.27)	712.75 (623.59–832.12)	0.892 ^¥^
Calcium (mg/dL)	8.93 ± 0.57	9.18 ± 0.55	0.091 ^†^
Parathormone (pg/mL)	48.25 ± 19.57	38.71 ± 12.32	0.051 ^†^
Vitamin D (ng/mL)	14.3 (8.45–17.5)	26.25 (21–32)	<0.001 ^¥^
Immature granulocyte (IG) (%)	0.2 (0.2–0.3)	0.2 (0.2–0.3)	0.918 ^¥^
White blood cell (10^3^/µL)	7.1 (5.8–9.1)	6.42 (5.9–8.17)	0.389 ^¥^
Red blood cell (10^6^/µL)	4.7 (4.5–4.9)	4.6 (4.25–5)	0.258 ^¥^
Hemoglobin (g/dL)	13.38 ± 1.43	13.6 ± 1.49	0.539 ^†^
Hematocrit (%)	41.5 ± 2.34	40.55 ± 3.9	0.264 ^†^
Platelet (10^6^/µL)	200.5 (180–223.5)	226 (210–259)	0.004 ^¥^
Lymphocytes (10^3^/µL)	2.65 (2.25–3.1)	2.44 (2.04–2.6)	0.107 ^¥^
Lymphocytes (%)	29.7 (27.5–34.5)	32 (28–33.6)	0.662 ^¥^
Neutrophil (10^3^/µL)	3.95 (3–5.45)	2.98 (2.57–3.31)	<0.001 ^¥^
Neutrophil (%)	54.01 ± 7.8	29.8 ± 3.91	<0.001 ^†^
Monocyte (10^3^/µL)	0.6 (0.5–0.72)	0.5 (0.42–0.6)	0.013 ^¥^
Monocyte (%)	7.2 (6.4–8.9)	7.6 (6.8–8.4)	0.747 ^¥^
Eosinophil (10^3^/µL)	0.21 (0.11–0.32)	0.15 (0.1–0.2)	0.094 ^¥^
Eosinophil (%)	1.8 (1.2–3.1)	2 (1.5–2.9)	0.765 ^¥^
CRP (mg/L)	3.3 (2–5.35)	1.18 (0.68–1.7)	<0.001 ^¥^
Neutrophil lymphocyte ratio (NLR)	1.66 ± 0.52	1.29 ± 0.37	0.001 ^†^
Platelet lymphocyte ratio (PLR)	78.04 (62–94.74)	98 (85.6–117.96)	0.003 ^¥^
Systemic immune inflammation index (SII)	335.6 (227.89–425.03)	314.22 (227.5–365.69)	0.389 ^¥^

^†^: Independent samples *t*-test; ^¥^: Mann–Whitney U-test.

**Table 3:** The descriptive data of otolin-1 in the case and control groups.

		Case	Control
Otolin-1	mean ± std	685.85 ± 202.72	307.88 ± 110.43
	Median (25 p–75 p)	710.44 (584.35–837.39)	280.45 (212.7–419.61)
	Median (Min–Max)	710.44 (307.17–997.37)	280.45 (159.31–523.51)

**Table 4:** The results of ROC analyses for markers of benign paroxysmal positional vertigo.

Variables	AUC	95 CI of AUC	*p*-Value	Cutoff	Sensitivity	Specificity
Otolin-1	0.933	0.881–0.986	<0.001	525 ^¶^	79.17%	100%
CRP	0.867	0.787–0.947	<0.001	2 ^¶^	79.17%	80.77%
				2.2 ^¶^	68.75%	96.15%
NLR	0.705	0.587–0.823	0.004	1.65 ^¶^	50.00%	84.62%
PLR	0.713	0.588–0.838	0.003	87.5 *	72.92%	73.08%
Vitamin D	0.913	0.848–0.979	<0.001	20 *	83.33%	88.46%

^¶^ If greater than or equal to. * If less than or equal to.

**Table 5:** Correlations of otolin-1 and otocolin-90 with other hematological parameters.

All Group			
		Otoconin-90 (ng/L)	Vitamin D
Otolin-1 (pg/mL)	r	0.393	−0.682
	*p*	0.001	<0.001
Otoconin-90 (ng/L)	r		−0.404
	*p*		<0.001
Patients			
		Otoconin-90 (ng/L)	Vitamin D
Otolin-1 (pg/mL)	r	0.693	−0.586
	*p*	<0.001	<0.001
Otoconin-90 (ng/L)	r		−0.672
	*p*		<0.001
Control			
		Otoconin-90 (ng/L)	Vitamin D
Otolin-1 (pg/mL)	r	0.039	0.181
	*p*	0.851	0.376
Otoconin-90 (ng/L)	r		−0.151
	*p*		0.460

## Data Availability

The data underlying this article are available in the article. If needed, please contact the corresponding author. The email address is huzun59@hotmail.com.
